# Case Report: Anlotinib combined with PD-1 inhibitor and sequential GA regimen or FOLFIRINOX Chemotherapy in treatment of KRAS G12V mutated pancreatic ductal adenocarcinoma with liver metastasis: A case and literature review

**DOI:** 10.3389/fimmu.2022.1016647

**Published:** 2022-10-13

**Authors:** Yunpeng Wang, Bofang Wang, Lin Xiang, Junge Deng, Bo Xu, Puyi He, Weigao Pu, Haiyun Wang, Yong Fan, Hao Chen

**Affiliations:** ^1^ Second Clinical Medical College, Lanzhou University, Lanzhou, Gansu, China; ^2^ Department of Surgical Oncology, Lanzhou University Second Hospital, Lanzhou, China; ^3^ Department of General Surgery, Lanzhou University Second Hospital, Lanzhou, China; ^4^ Key Laboratory of the Digestive System Tumors of Gansu Province, Lanzhou, China; ^5^ Department of Cancer Center, Lanzhou University Second Hospital, Lanzhou, China

**Keywords:** Anlotinib, PD-1 inhibitor, chemotherapy, metastatic pancreatic ductal adenocarcinoma, KRAS, case report

## Abstract

There is a high mortality rate associated with pancreatic cancer, and the incidence has been rising globally in recent decades. When patients are diagnosed, there is little chance that surgery will be beneficial. Systemic chemotherapy is the currently accepted treatment option for patients with metastatic advanced pancreatic cancer. However, a very limited survival improvement is possible with chemotherapy for advanced pancreatic cancer, and chemotherapy resistance plays a significant role in poor prognosis. Despite the fact that targeting growth factor receptor inhibitors such as anti-vascular endothelial growth factor (VEGFR) antibodies significantly improves survival in pancreatic cancer, only a very small number of patients benefit from the treatment. As emerging drugs, immune checkpoint inhibitors (ICIs) have demonstrated significant therapeutic effects in several tumor types, but monotherapy is not effective in pancreatic cancer. In the first-line treatment of solid tumors, combination therapy may result in remarkable outcomes. Here in, we have reported a younger patient with pancreatic ductal adenocarcinoma with liver metastasis (PDACLM) who had a long-term partial response and good tolerance to the combination of anlotinib and programmed cell death protein 1 (PD-1) inhibitor and chemotherapy. Gene analysis suggested only one mutation in the Kirsten rat sarcoma viral oncogene (KRAS) G12V gene. Consequently, there is some hope for patients with pancreatic cancer, especially for KRAS G12V gene mutated patients. Upon reviewing the literature, this patient’s combination therapy is the first to have been reported.

## Introduction

Pancreatic ductal adenocarcinoma (PDAC) is a highly lethal malignancy with a 5-year survival rate of <9%. Global statistics predict that the prevalence of PDAC will nearly double by 2040 (1), placing it as the fourth leading cause of cancer-related deaths. Despite the fact that a lot of research has been conducted on diagnosis and treatments over the past two decades, it is estimated that fewer than 7% of patients with PDAC will survive 5 years after diagnosis (2). The liver is the most common metastatic site of pancreatic cancer. Compared with lung metastasis and bone metastasis, the prognosis of pancreatic cancer patients with liver metastasis is the worst (3), The median survival of patients with metastases is only 3-6 months, and the 5-year overall survival (OS) rate is less than 4% (4). Accordingly, the pretty low possibility of radical surgery and lacking an effective treatment make pancreatic cancer with liver metastasis a dismal prognosis.

The treatment of advanced pancreatic cancer, including chemotherapy, immunotherapy, targeted therapy, and radiotherapy, has only made modest progress over the past decade (5–7). Patients with advanced diseases remain mainly treated with chemotherapy and topical surgical treatment including surgical resection or radiofrequency ablation depending on the site number and size of the metastases. In accordance with the 2020 guidelines of the National Comprehensive Cancer Network, first-line chemotherapy regimens for advanced pancreatic cancer (PDACLM) include GA regimen (gemcitabine, albumin-bound paclitaxel) and FOLFRINOX (oxaliplatin, irinotecan, fluorouracil, and leucovorin). There is, however, little survival benefit and significant toxicity associated with cytotoxic chemotherapy regimens. PDAC has shown refractoriness to mono-immunotherapy, despite immunotherapy’s success rates in other solid organ tumors (8). Anti-tumor microenvironment-targeted therapy is also a promising therapeutic strategy. However, the tumor microenvironment of PDACLM is extremely complex and contains multiple interlaced signaling pathways. So far, the most relevant targets for tumor progression and metastasis have not yet been found. As our understanding of tumor biology and genetic alteration of PDACLM grows, targeted treatment options become more readily available Liver metastasis is mainly related to blood vessel dissemination so the use of vascular-targeted drugs is theoretically feasible, and the decline of the local immune surveillance function promotes the metastasis, therefore the target and immunotherapy are theoretically supported but it is still early in the evaluation process of the clinical impact of these regimens. In this way, combination strategies may change the paradigm of PDACLM treatment.

Here, we report a case of a PDAC patient with multiple hepatic metastases who had a better response and good tolerance to a quadruple combination of standard chemotherapy, PD-1 blockade, and anlotinib. As of now, this is a wonderful report that shows that the therapeutic strategy can be both safe and effective, it is hoped that this preliminary finding will be confirmed in the next cohort of patients, giving patients with this lethal condition a glimmer of hope.

## Case Presentation

**Figure 1:**
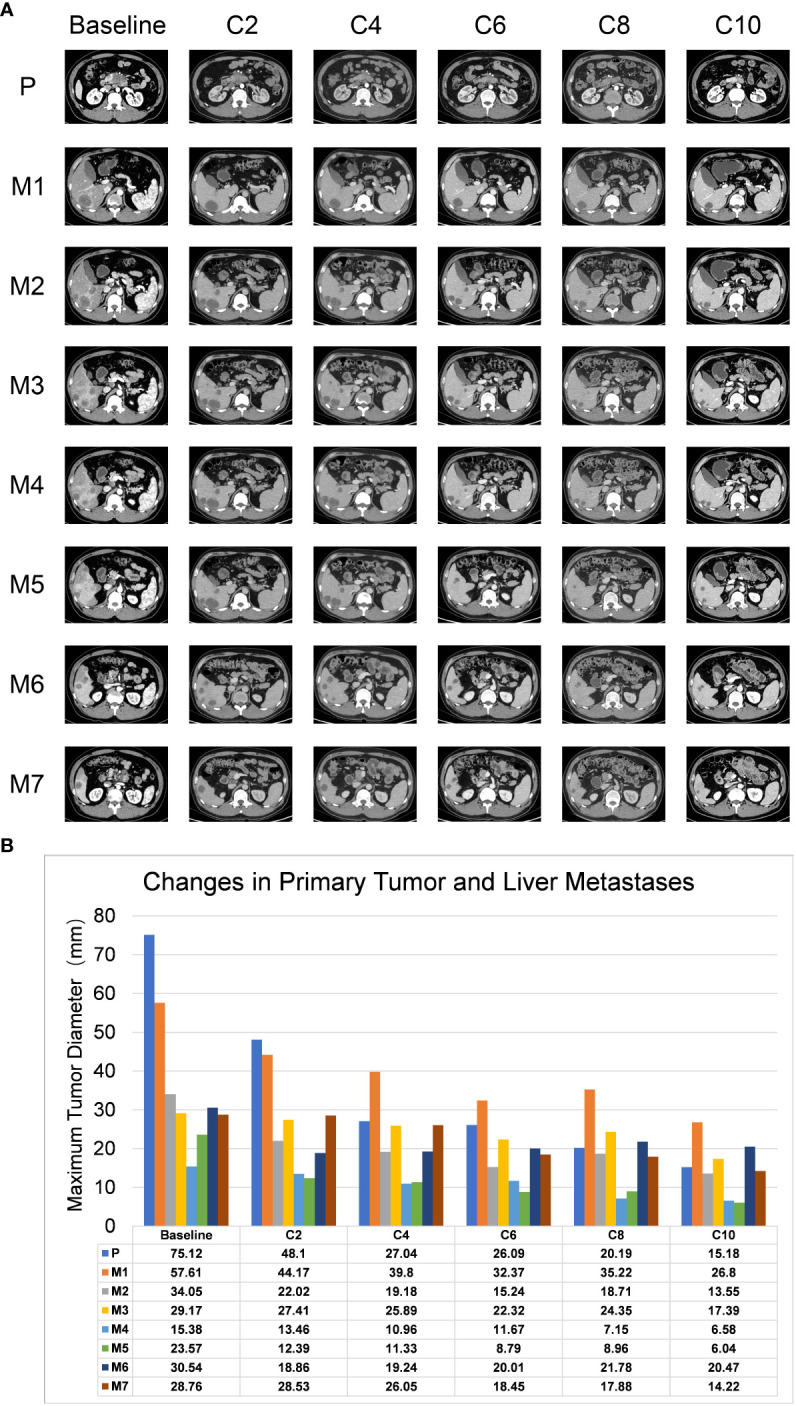
Representative images of CT scans for lesions. **(A)** CT scans showed changes in primary and metastatic lesions after combination therapy. C2-C10 represents the re-examination every two cycles, P represents the primary lesions, and M1-M7 represents the 7 liver metastases. **(B)** The histogram demonstrated the change trends in primary tumor and liver metastases, including maximum tumor diameter (Y-axis) corresponding to the treatment timeline. The X-axis shows the treatment circle.

**Figure 2:**
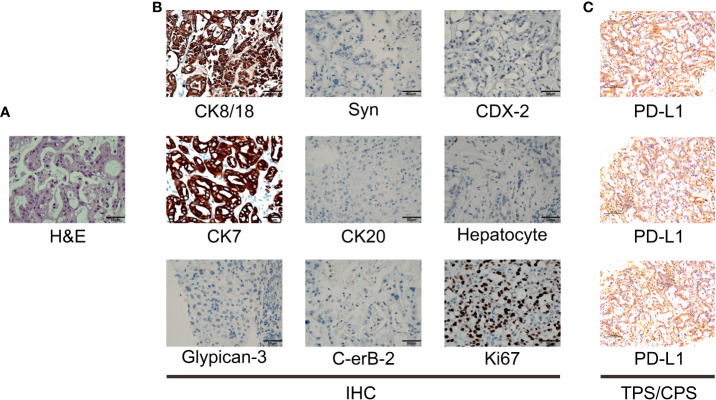
Pathological examination results. **(A)** The hematoxylin-eosin (H&E) staining in the microscopic observation (400×). **(B)** IHC of liver metastases of pancreatic ductal adenocarcinoma showed CK8/18+, Syn-, CDX-2-, CK7+, CK20-, Hepatocyte-, Glypican-3-, C-erB-2(0), Ki67 (60%+), original magnification ×400. **(C)** PD-L1 IHC staining of hepatic metastases tested that the Tumor cell Proportion Score (TPS) is 2-4, and the Combined Positive Score (CPS) is 2-4.

**Figure 3:**
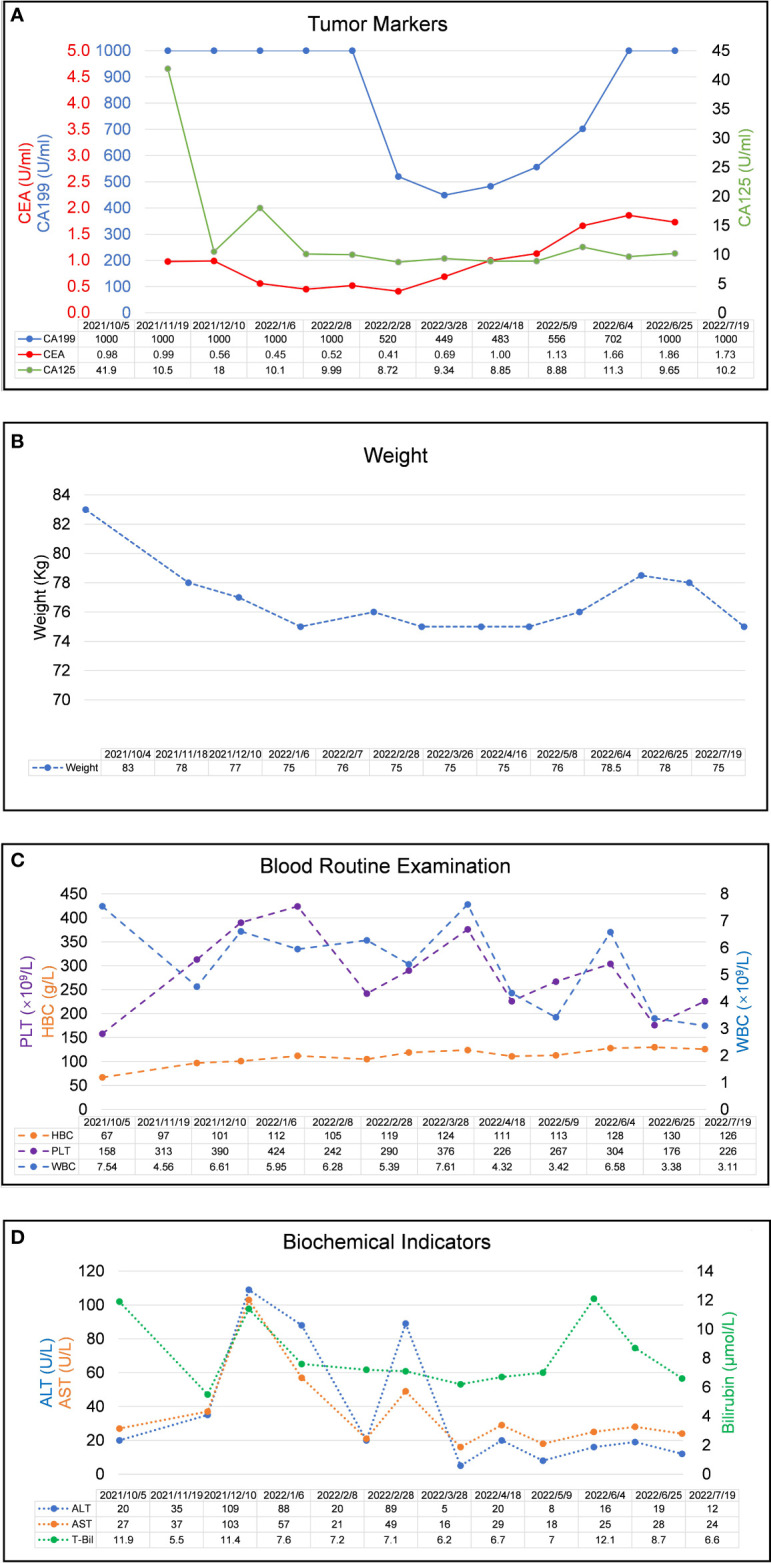
Laboratory examination results during the clinical course. **(A)** Trends in the level of tumor markers, including CA 199, CA125, and CEA (right Y-axis) correspond to the treatment timeline. **(B)** Trends in the level of weight changes corresponding to the treatment timeline. **(C)**. Trends in the level of blood routine examination, including PLT, HBC, and WBC (right Y-axis) correspond to the treatment timeline. **(D)** Trends in the level of biochemical indicators, including ALT, AST, and Bilirubin (right Y-axis) correspond to the treatment timeline. All X-axes show the date of disease progression. (CEA, carcinoembryonic antigen; CA199, carbohydrate antigen 19-9; CA125, carbohydrate antigen 125, WBC, white blood cell count; HBC, hemoglobin; PLT, blood platelet count, ALT, alanine aminotransferase; AST, aspartate aminotransferase).

**Figure 4:**
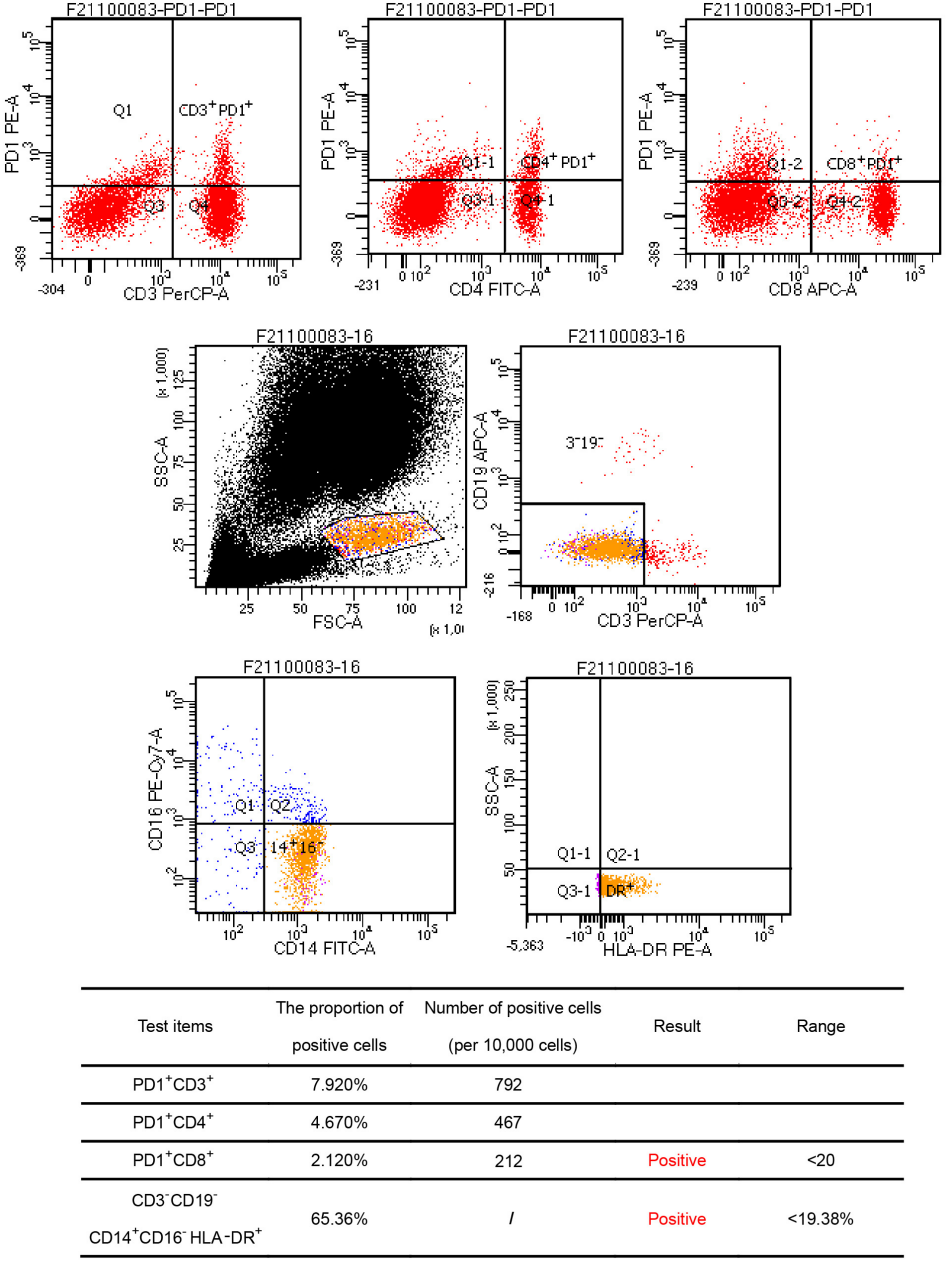
Flow cytometry results. Findings showed the number of PD1^+^CD8 positive cells was 212/10,000; The proportion of CD3^-^CD19^-^CD14^+^CD16^-^HLA-DR positive cells was 65.36%, and all the above cell numbers were positive.

A 44-year-old Asian male patient was admitted to our hospital due to “Upper abdominal pain for more than 1 month”. This patient had intermittent nausea and vomiting after food ingestion. The patient has not received any other treatment in the past and has no family history of cancer. Abdominal enhanced computed tomography (CT) scan showed space-occupying lesions in the neck of the pancreas, multiple enlarged lymph nodes around the pancreas and retroperitoneum, accompanied by multiple space-occupying lesions in the right lobe of the liver, considered to be pancreatic cancer with liver metastases (**Figure 1A**). A Chest CT scan revealed no obvious abnormality. An examination of the whole body’s bone scintigraphy did not reveal any abnormalities. Ultrasound of lower extremity vessels showed thrombosis in bilateral popliteal and right peroneal veins. Later a liver biopsy was confirmed to be a moderately to poorly differentiated adenocarcinoma (**Figure 2A**). Immunohistochemical (IHC) results showed CK8/18+, Syn-, CDX-2-, CK7+, CK20-, Hepatocyte-, Glypican-3-, C-erB-2(0), Ki67 (60%+), (**Figure 2B**) and serum tumor markers test were abnormal, carbohydrate antigen 125 (CA125): 41.9U/ml, carbohydrate antigen 19-9 (CA199) >1000U/ml (**Figure 3A**). The coagulation test showed that the prothrombin time was 13.6 seconds, the prothrombin activity was 72%, and the international normalized ratio was 1.24. The immune checkpoint expression test report showed that the number of PD1^+^CD8 positive cells was 212/10,000; The proportion of CD3^-^CD19^-^CD14^+^CD16^-^HLA-DR positive cells was 65.36%, detected by a peripheral blood cell flow cytometer (**Figure 4**). Genetic testing indicated that the patient had KRAS G12V gene mutation, the tumor mutation burden (TMB) value was 5.16, the microsatellite test was stable (MSS), and no pathogenic DNA mismatch repair (MMR) gene mutation was detected. The programmed cell death ligand 1 (PD-L1) expression results are Tumor Proportion Score (TPS) (2-4%), and Combined Positive Score (CPS) (2–4) (**Figure 2C**). This patient’s primary diagnosis was pancreatic ductal adenocarcinoma with liver metastasis and thrombus in right lower extremity. Furthermore, the patient’s Tumor-Node-Metastasis (TNM) stage, cT3N1M1, Karnofsky score, 80 points, Eastern Cooperative Oncology Group (ECOG) score, 1 point, visual analogue scale (VAS) score,6 points, and nutrition risk screening (NRS), 3 points.

**Figure 5:**
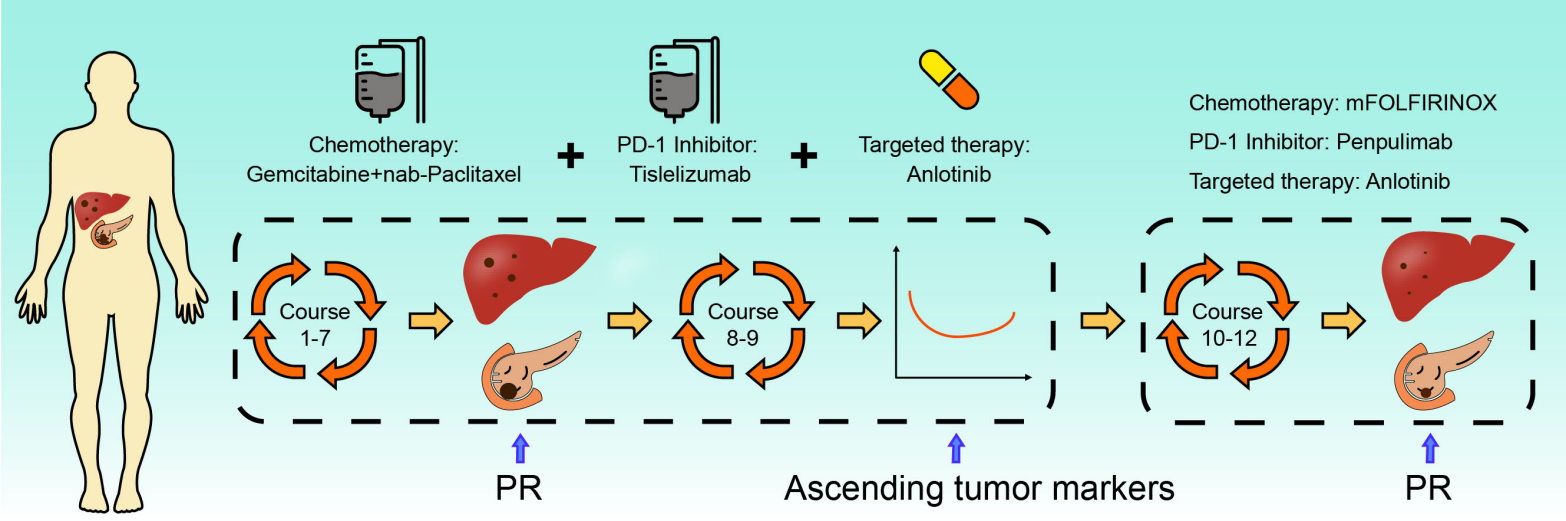
The schematic diagram for the patient timeline information.

Members of our multidisciplinary team (MDT) convened to discuss the treatment and workup plan for this patient and suggested that the patient can be treated with anlotinib in combination with gemcitabine and nab-Paclitaxel (GA) and Tislelizumab (a PD-1 inhibitor) regimen. And then specifically 1000mg/m^2^ gemcitabine and 125 mg/m^2^ nab-paclitaxel on days 1 and 8 (the patient’s body surface area is 2.07 m^2^), Tislelizumab (BeiGene Company) 200mg intravenous infusion and anlotini (Chia Tai Tianqing Company) 12 mg orally for 2 weeks were administrated in every treatment circle for 21days. Before antitumor therapy, we also performed antithrombotic therapy (enoxaparin sodium 0.4ml per day). After half a month of antithrombotic therapy, the patient’s coagulation function gradually returned to normal. After 3 months, the ultrasonography of the lower extremity blood vessels showed that the blood flow in the bilateral arteries was smooth, and there was no thrombosis in the veins. Routinely CT assessments were performed every two cycles of treatment and abdominal ultrasonography between two circles when CT is not performed. Repeated CT scan examination pictured the patient’s primary lesions were dramatically shrinking, compared to the last CT-scan results, (**Figure 1A**), however, there was little change in liver metastases during the first two cycles of treatment. Continuing treatment, the patient’s primary lesions, and liver metastases continue to shrink over the same period. Meanwhile, the patient’s tumor markers CA-199 gradually decreased after 5 cycles of treatment (reduced from >1000U/ml to 520U/ml) and reached the nadir on the sixth cycle (446U/ml). However, after 9 cycles of treatment, the patient’s tumor markers CA-199 doubled from its previous nadir (**Figure 3A**) and the primary lesion has grown larger than the previous one. Therefore, after the second MDT evaluation and discussions of the patient’s condition, during the 10th cycle of treatment, the GA program was switched to mFOLFIRINOX, (Oxaliplatin: 68 mg/m^2^, Irinotecan:135mg/m^2^, Leucovorin:400mg/m^2^, 5-Fluorouracil:2400mg/m^2^), mFOLFIRINOX chemotherapy is administered every 3 weeks, and Tislelizumab was switched to Penpulimab (Chia Tai Tianqing Company):200mg, anlotinib was used according to the original plan. Surprisingly, after 1 cycle of new treatment regimens as mFOLFIRINOX, CT scans illustrated that the primary lesion was significantly smaller than in the previous cycle, and the liver metastases were decreasing (**Figure 1A**). Histograms show trends in primary tumors and liver metastases and exact values for each stage (**Figure 1B**). Before the 12th cycle of medication, we performed abdominal ultrasonography and found that the structure of the pancreas was normal and the primary lesions disappeared (**Table 1**). Even more encouraging was the patient’s absence of harmful side-effect during treatment, and his excellent adherence to therapy. The patient’s liver function impairment, thyroid function, and pancreatic function were basically normal (**Figure 3D**). The most serious treatment-related adverse events (TRAEs) of this patient throughout the clinical course were grade 2 leukocytopenia and grade 2 anemia, which was recovered under drug intervention before the next cycle of treatment (**Figure 3C**). In general, he demonstrated good tolerance and did not suffer any toxicities of grade 3 or higher. After a series of treatments, symptoms such as nausea, vomiting, and abdominal pains stopped. Furthermore, post-treatment, this patient resumed a normal diet, and his quality of life improved tremendously. Patients have greater confidence in subsequent treatment. Up to now, the patient’s weight has remained basically stable (**Figure 3B**). Currently, the patient remains at our health center for treatment and monitoring, it has been over 10 months since this patient received his or her first treatment, and his progression-free survival (PFS) is more than 10 months. The patient’s whole course of treatment is shown in (**Figure 5**)

**Table 1:** Changes of primary lesions and liver metastases by Abdominal unstrasound.

Date	C1	C3	C5	C7	C9	C11
Primary (cm)	2.5*2.1	3.0*2.4	4.5*2.2	2.2*1.7	1.9*1.3	Normal
Mmax (cm)	4.0*4.2	3.7*3.4	4.0*3.2	2.9*2.8	2.5*2.5	2.2*2.3

C1-C11: The number of cycles of treatment.

Primary: The size of the primary lesion; Mmax; The size of the largest liver metastases.

## Discussion and conclusions

The prognosis of pancreatic cancer is poor because the cancer is aggressive, develops rapidly, and is highly invasive. Pancreatic cancer is currently treated in a less than ideal way, and the median survival time is just six months (9). However, for unresectable locally advanced pancreatic cancer or pancreatic cancer with distant metastasis, the overall treatment effect is poor, and relevant clinical research is recommended.

In tumor cells, albumin-bound paclitaxel is distributed directly by binding to albumin receptors attached to the surface of vascular endothelial cells, which increased concentrations of paclitaxel in the interstices of tumor cells can enhance antitumor effectiveness, and is safe and low toxic (10). First-line treatment resulted in a median PFS of 5.5–6.4 months, and median OS of 8.7–11.1 months (11, 12). Second-line PFS and OS were 3.0–3.2 months and 6.1–6.6 months, respectively (13, 14). According to the randomized phase III MPACT trial, 861 patients with stage IV pancreatic cancer were treated with nab-paclitaxel and gemcitabine combined as first-line therapy. The overall survival rate of patients who had a combination treatment increased from 6.6 months to 8.7 months (P <0.001) (12, 15). The objective response rate was 23% in the combination group versus 8% in the gemcitabine group (P < 0.001). Several phase III trials also have shown that patients with metastatic pancreatic cancer may benefit from gemcitabine plus nab-paclitaxel compared with gemcitabine (12, 16, 17). As systemic chemotherapy prolongs median survival times from 2-4 months, it also causes many adverse reactions that affect adherence to medication (12). A clinical practice (18) of FOLFIRINOX for first-line treatment of advanced pancreatic cancer showed that liver metastases (p = 0.019; hazard ratio, HR, 0.59, 95% confidence interval, CI, 0.380.96) were associated with poorer OS in multivariate analysis. Cancers of the pancreas remain poorly prognosticated long-term. Given the lack of effective treatment, improved therapeutic strategies are greatly needed to improve the clinical outcomes of patients with PDACLM.

Anti-angiogenic drugs have been researched and developed extensively in recent years and pancreatic cancer patients are increasingly turning to anti-angiogenic therapy to control tumor growth and progression. Studies have found that patients with pancreatic cancer are more likely to have liver metastasis when their vascular endothelial growth factor (VEGF) levels are high in cancer tissue, which indicates a poor prognosis (19). The clinical phenomenon of liver metastasis in pancreatic cancer suggests that tumor cells use anatomical characteristics to transfer through blood vessels, so targeting drugs for blood vessels theoretically act on both primary and metastatic lesions. Anlotinib is an orally available multi-targeted receptor tyrosine kinase inhibitor able to inhibit both VEGFR, Fibroblast Growth Factor Receptor 1-4 (FGFR1-4), Platelet-Derived Growth Factor Receptor Alpha (PDGFR-α), Platelet-Derived Growth Factor Receptor Beta (PDGFR-β), c-kit proto-oncogene protein (c-Kit), and RET proto-oncogene (Ret), as well as act on tumor blood vessels, it can also inhibit tumor cell growth (20). Clinical studies (21) have shown that anlotinib is effective and significantly reduces tumor burden in various solid tumors, including colon adenocarcinoma, non-small cell lung cancer, renal clear cell carcinoma, medullary thyroid carcinoma, and soft tissue sarcoma. Some studies found that anlotinib showed tumoricidal effects in pancreatic cancer cells both *in vivo* and *in vitro*, and demonstrated that reactive oxygen species-induced endoplasmic reticulum stress is a novel mechanism by which anlotinib causes apoptosis in pancreatic cancer cells (22). Identified targetable pathway as described above indicated that anlotinib may be a rational option for PDACLM (23).

Moreover, synergistic effects are observed when the anti-angiogenesis drug is combined with chemotherapy (24, 25). Nevertheless, most studies to date have only examined anlotinib monotherapy treatments (26, 27). Thus, future studies are needed to evaluate the combination of anlotinib and other therapies. A retrospective study (28) included 33 patients with advanced pancreatic cancer, of which 17 received anlotinib in combination with GA regimen (anlotinib combined therapy group) and 16 received GA alone (chemotherapy group). The results of the study showed that compared with chemotherapy alone, the anlotinib combination treatment group had significantly improved PFS and OS. In addition, we are particularly pleased to observe that comparing the 3-month PFS rate, 6-month PFS rate, 6-month OS rate and 12-month OS rate of the two groups of patients, the anlotinib combination treatment group was significantly higher than the chemotherapy group alone. Median PFS was 5.0 months (95% CI: 4.97–5.94 months) in the anlotinib-combination arm, compared with only 2.7 months (95% CI: 2.4–3.3 months) in the chemotherapy-only arm (P=0.022). Median OS was 9.0 months (95% CI: 6.55–11.45 months) in the anlotinib-combination arm compared with 6.0 months (95% CI: 1.08–10.92 months) in the chemotherapy alone arm (P =0.006). Based on this, we decided to add anlotinib to the conventional GA regimen to explore whether the combination therapy can bring better survival benefits to patients at an advanced stage of pancreatic cancer.

In recent years, cancer patients have benefited greatly from immunotherapy with immune checkpoint inhibitors. T cells are activated and immunosuppression is blocked, allowing T cells to play an immune role. There is growing evidence that PD-1 inhibitors enhance antitumor activity and improve survival in various malignant tumors (29–31). Anti-PD-1 monotherapy alone failed to bring significant benefits to patients with advanced pancreatic cancer, according to additional studies (32, 33). It is speculated that PD-1/PD-L1 blockers are limited in their efficacy because of two main reasons. First, it is immune suppression caused by a high tumor burden that makes PD-1/PD-L1 blockade ineffective in treating pancreatic cancer. Second, pancreatic cancer does not exhibit immunogenic properties (34). Consequently, PDACs are considered to be resistant to single-agent immunotherapy with the exception of those with a high level of microsatellite instability (MSI-H) or lacking mismatch repair deficient (dMMR) (35). The synergistic anticancer effects of combining immunotherapy with chemotherapy are however promoted by the fact that many chemotherapeutic medications have immunostimulatory properties (35–37). Tumor antigens can be released from cancer cells as a result of chemotherapy, and anticancer immune responses can be reactivated to suppress tumor growth as a result of chemotherapy (15). Combining immunotherapy with chemotherapy is expected to enhance either monotherapy’s antitumor effects (36). Furthermore, some studies have indicated that blocking VEGFR2 does not affect T cell infiltration and immune activation induced by blocking PD-1, there is a synergistic anti-tumor effect *in vivo* when PD-1 and VEGFR2 are both blocked simultaneously (38–41). Clinical studies have revealed that anlotinib can enhance the therapeutic effect of PD-1 inhibitors, so it is believed that anlotinib has a significant synergistic effect with PD-1/PD-L1 checkpoint blockers (39). A study by Yang et al. showed that anlotinib and anti-PD-1/PD-L1 antibody synergistically promoted natural killer cell infiltration to provide therapeutic benefits, M1-like tumor-associated macrophages (TAM), and dendritic cells while reducing the infiltration of M2-like TAM (42). As reported in another study, anlotinib reduces PD-L1 expression on vascular endothelial cells through the inactivation of the AKT pathway, causing an increase in the CD8/FoxP3 ratio within the tumor immune microenvironment (43). As a result of anlotinib’s effect on the immunosuppressive tumor microenvironment, the antibody against PD-1/PD-L1 appears to be more effective. As a result of multiple immune-suppressive components being activated in the tumor microenvironment, immunotherapy in solid tumors is limited (44). It has been shown that VEGF-VEGFR signaling contributes to local and systemic immunosuppression in a variety of ways. It has been shown that excessive activation of the VEGF-VEGFR pathways affects the trafficking of immune cells to tumors as well as the upregulation of intercellular adhesion molecule-1(ICAM-1) and vascular cell adhesion molecule-1 (VCAM-1) expression (45). Also, VEGF stimulates various mechanisms to reprogramme the immunosuppressive microenvironment, the immune system can be suppressed by increasing immunosuppressive cytokines (IL-10, TGF-b), enhancing inhibitory checkpoints (such as PD1, CTLA4, and LAG-3), and increasing the presence of MDSCs and Treg (46, 47). Simultaneously, in this case, according to the TMB value (5.16), TPS (2-4%), and CPS (2–4) as well as the patient’s peripheral immune blood, we learned and noted that the immune checkpoints PD1^+^CD8, CD3^-^CD19^-^CD14^+^CD16^-^HLA-DR were all positive. In addition, CD3^-^CD19^-^CD14^+^CD16^-^ the proportion of HLA-DR monocyte subsets in peripheral blood were significantly positively correlated with PD-1 blockade treatment effect and overall survival, and patients with high positive expression had better PD-1 treatment effect and longer overall survivals. As mentioned above, we decided to add anlotinib and Tislelizumab to the conventional GA regimen to explore whether the four-drug combination can bring better survival benefits to patients at an advanced stage of pancreatic cancer.

There is a wide range of somatic mutations observed in different types of malignant tumors. It is estimated that each specimen of pancreatic ductal adenocarcinoma contains between 100 and 150 somatic mutations (48). Oncogene mutations in KRAS are present in nearly all PDAC specimens. There is also a high rate of p53 mutations (35%) and CDKN2A mutations (31%-55%), although these mutations are more common in later-stage tumors and metastatic sites than in primary tumors (49). Several other somatic mutations were detected in 5% to 10% of tested tumors, including ARID1A, ROBO2, KDM6A, PREX2, RNF43, EphA2, SHH, and INK4A/ARF (49–52). As a tumor suppressor, SMAD4 is unusual among other cancer types since it is seldom mutated (53). X6 Since 5–10% of all pancreatic cancers are estimated to be attributable to inherited risk factors, some familial syndromes with the genomic mutation are considered to increase the generation of PDAC (54). For example, The Peutz-Jeghers syndrome, resulting from a mutation in the tumor suppressor STK11 (also known as LKB1), results in a 35% increased risk of developing pancreatic cancer. Similarly, hereditary breast-ovarian cancer syndrome, most commonly attributed to mutations in BRCA1 or BRCA2, is associated with an increased risk of developing this type of cancer (55). In this article, we mainly discuss the KRAS gene mutation.

With regard to a KRAS (G12V) gene mutation, studies have shown that the mutation frequency of the KRAS gene in pancreatic cancer is high, about 61% (48). And the most common way of KRAS gene mutation is a point mutation, including KRAS-G12D mutation (41%), KRAS -G12V (28%), and KRAS-G12C (14%) mutation (18). The study by Takeshi et al. (56) showed that the KRAS genotype was associated with overall survival in pancreatic cancer patients. The overall survival of patients in the wild-type group was longer than that in the mutant group (479 days vs. 255 days) (P = 0.03). In addition, in the KRAS gene mutation group, the overall survival of patients with different mutation sites was also significantly different. The overall survival of patients with three common KRAS mutants G12D, G12V, and G12R were 242, 338, and 204 days, respectively. G12V mutant patients had longer overall survival than G12D (P = 0.01) or G12R (P < 0.01) mutant patients. The mutated KRAS oncogene can promote uncontrollable cell proliferation and malignant transformation through PI3K-PDK1-AKT and RAF-MEK1/2-ERK1/2 signaling pathways (57), thereby promoting tumor progression. Moreover, anlotinib may exert its anti-tumor effect mainly by blocking the VEGF/PI3K/AKT signaling pathway to inhibit tumor cell proliferation, invasion, and cell cycle-induced apoptosis. The study by Soukaina et al. (58) showed that the KRAS gene silencing strategy can reduce pancreatic tumor growth and enhance the chemotherapeutic effect of gemcitabine in pancreatic cancer. Although the relationship between KRAS gene mutation and the effect of target and immunotherapy for pancreatic cancer patients has not been fully clarified at present, further research is needed, but these previous studies can further prove the rationality of our regimens.

Chemotherapy targeted and immunotherapy will inevitably bring different degrees of adverse effects. In the ALTER0303 clinical trial, the most common ADRs in the anlotinib group included hypertension (64.6%), fatigue (46.3%), increased thyroid-stimulating hormone (44.6%), and hand-foot-skin reaction (43.2%) (59). The Keynote189 study (60) showed that the adverse reactions of grade 3 or higher with PD-1inhibitor combined with chemotherapy compared with chemotherapy alone were 71.9% and 66.8%. The incidence of immune-related adverse events (irAEs) of grade 3 or higher in the combination therapy group was 10.9%, and the higher incidence was pneumonia (3.0%), skin toxicity (2.2%), colitis (1.5%), and nephritis (1.5%). In this case, his impressive efficacy was accompanied by good tolerance to these quadruple anti-tumor drugs, especially PD-1 inhibitors whose immune-related adverse events should not be underestimated. During treatment, he encountered grade 1 myelosuppression, and grade 2 anemia, which was successfully treated with recombinant human interleukin-11 and recombinant human erythropoietin injection. Subsequently, he developed no more significant toxicities and tolerated a total of 10 cycles of the combination therapy. His thyroid function, liver, and kidney function were still within the normal range.

According to this case report, chemotherapy, targeted immunotherapy, may be a safe, extremely effective method of treating pancreatic cancer with liver metastasis. Since different drugs may interact through multiple mechanisms, combination therapy has great complexity. How to conduct tumor combination therapy more rationally and effectively and with low toxicity still needs to be further explored. In addition, this study has some limitations, including the fact that only one case was evaluated, making it necessary to test this therapeutic treatment strategy in more cases. In order to confirm the efficacy and safety of this regimen in treating patients with advanced stages of pancreatic cancer, more clinical trials and high-quality mechanisms studies are needed.

## Data availability statement

The original contributions presented in the study are included in the article/Supplementary Material. Further inquiries can be directed to the corresponding authors.

## Ethics statement

The studies involving human participants were reviewed and approved by Ethics Committee of the Second Hospital of Lanzhou University. The patients/participants provided their written informed consent to participate in this study.

## Author contributions

HC, YF, and YW designed this study. YW and BW wrote the manuscript. PH and WP participated in the coordination of the study and interpretation of the results. LX and JD analyzed the data. BX and HW revised the manuscript. All authors contributed to the article and approved the submitted version.

## Funding

This study was supported by National Natural Science Foundation of China (82160129,82203267) and Key Talents Project of Gansu Province (No.2019RCXM020) and Natural Science Foundation of Gansu Province (20YF3F033) and Key Project of Science and Technology in Gansu province(19ZD2WA001) and Natural Science Foundation of Gansu Province (22JR5RA945) and Science and technology project of Chengguan District of Lanzhou City (2019RCCX0034) and Cuiying Scientific and Technological Innovation Program of Lanzhou University Second Hospital (No. CY2017-ZD01).

## Acknowledgments

Thanks, Ewetse Paul Maswikiti for revising the syntax in this article.

## Conflict of interest

The authors declare that the research was conducted in the absence of any commercial or financial relationships that could be construed as a potential conflict of interest.

## Publisher’s note

All claims expressed in this article are solely those of the authors and do not necessarily represent those of their affiliated organizations, or those of the publisher, the editors and the reviewers. Any product that may be evaluated in this article, or claim that may be made by its manufacturer, is not guaranteed or endorsed by the publisher.
